# One-step individual patient data (IPD) network meta-analysis of survival data using royston-parmar models

**DOI:** 10.1186/1745-6215-16-S2-P161

**Published:** 2015-11-16

**Authors:** Suzanne Freeman, James Carpenter, Jayne Tierney

**Affiliations:** 1 MRC Clinical Trials Unit at UCL, London, UK; 2 London School of Hygiene & Tropical Medicine, London, UK

## Background

Network meta-analysis (NMA) combines direct and indirect evidence from trials to calculate treatment effect estimates and rank treatments. Modelling approaches for continuous and binary outcomes are relatively well developed, but less work has been done with time-to-event outcomes. Such outcomes have usually been analysed using Cox proportional hazard (PH) models, but in oncology with longer follow-up of trials, and time-dependent effects of targeted treatments, this may no longer be appropriate. Royston-Parmar models, fitted using WinBUGS, are a flexible alternative.

## Methods

Motivated by IPD from 39 trials comparing combinations of surgery, radiotherapy and chemotherapy from 7636 women with cervical cancer (3184 events), we developed an IPD Royston-Parmar NMA model for overall and disease-free survival. We fitted this model in one-step using WinBUGS. By including a treatment-log(time) interaction we were able to test for PH. Extensions allowed for covariate and treatment-covariate interactions.

## Results

A global test of PH showed it to be a reasonable assumption for these data. A joint test of the treatment-cancer stage interaction was significant. This was principally due to the differential effectiveness of chemoradiation versus radiotherapy by cancer stage at randomisation. The overall network-heterogeneity Q statistic was not significant, but there was evidence of moderate heterogeneity within one of the designs.

## Conclusion

Our approach provides a range of flexible models for one-step NMA of IPD survival data. It naturally handles missing covariate data. A practically important advantage is that the underlying Royston-Parmar model allows the approach to extend readily to handle non-PH in both treatment and treatment-covariate interactions.

